# Nail Gun Penetrating Renal Injury: A Case Report

**DOI:** 10.7759/cureus.22697

**Published:** 2022-02-28

**Authors:** Ali S Alothman, Ghassan I Alhajress, Alaa Elshaer, Saeed Bin Hamri

**Affiliations:** 1 Division of Urology, Department of Surgery, Ministry of National Guard - Health Affairs, Riyadh, SAU; 2 College of Medicine, King Abdullah International Medical Research Center, Riyadh, SAU; 3 College of Medicine, King Saud bin Abdulaziz University for Health Sciences, Riyadh, SAU; 4 Urology, Benha University, Benha, EGY

**Keywords:** nail extraction, occupational trauma, renal injury, renal salvage, penetrating renal trauma

## Abstract

The kidney is the genitourinary organ most affected by trauma, although the retroperitoneal location provides some protection. Renal injuries are classified according to the mechanism of trauma. Most of the penetrating renal injury cases in the literature are due to knife stabbing or handguns. We present an interesting case of a 22-year-old male with a penetrating renal injury caused by an electric nail gun. There was no report of a similar case in the literature.

## Introduction

Although the retroperitoneal location of the kidney gives some protection, it is the most injured genitourinary organ. Renal trauma occurs more frequently in younger age groups, with males the predominant gender. Renal injuries are classified according to the mechanism of trauma. The most frequently reported type of renal injury is blunt trauma, accounting for 71%-95% of all cases of renal injuries. Though penetrating renal injuries are less common, they are often associated with a less favorable prognosis and a higher risk of more serious interventions, such as a nephrectomy [1,2]. The mechanism through which penetrating renal injuries occur varies considerably, resulting in different grades of injury which may affect the management decision. Penetrating renal injuries are classified according to the velocity of the penetrating object. High and medium velocity injuries, caused by handguns and rifles, cause the most devastating and serious damage to the kidney, and low-velocity injuries such as knife stabbing can lead to a variable level of damage according to the location of the stab wound [2,3].

Most of the penetrating renal injury cases in the literature are due to knife stabbing or handguns. We present an interesting case of a 22-year-old male with a penetrating renal injury caused by an electric nail gun. There was no report of a similar case in the literature.

## Case presentation

**Figure 1:**
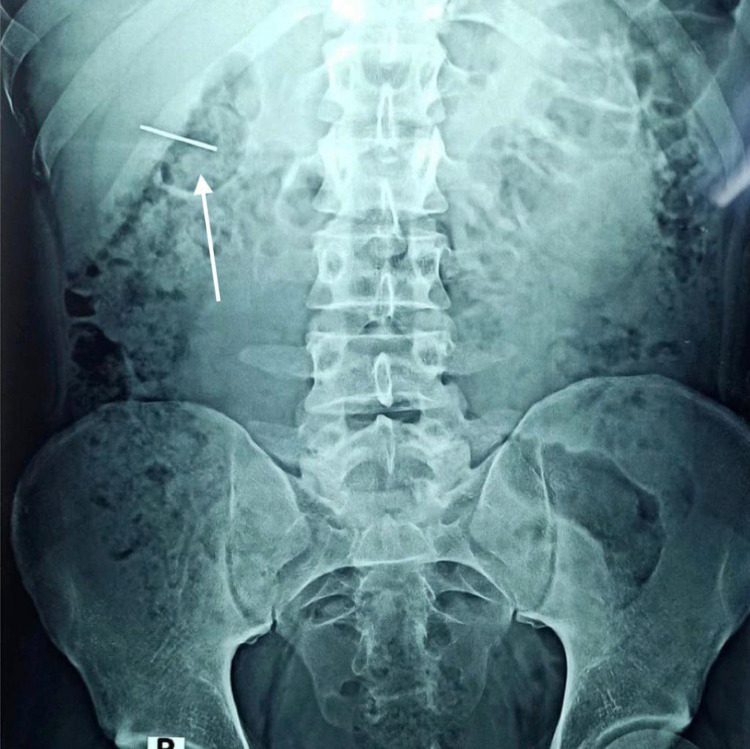
The metallic nail opposite the confinement of the right kidney.

**Figure 2:**
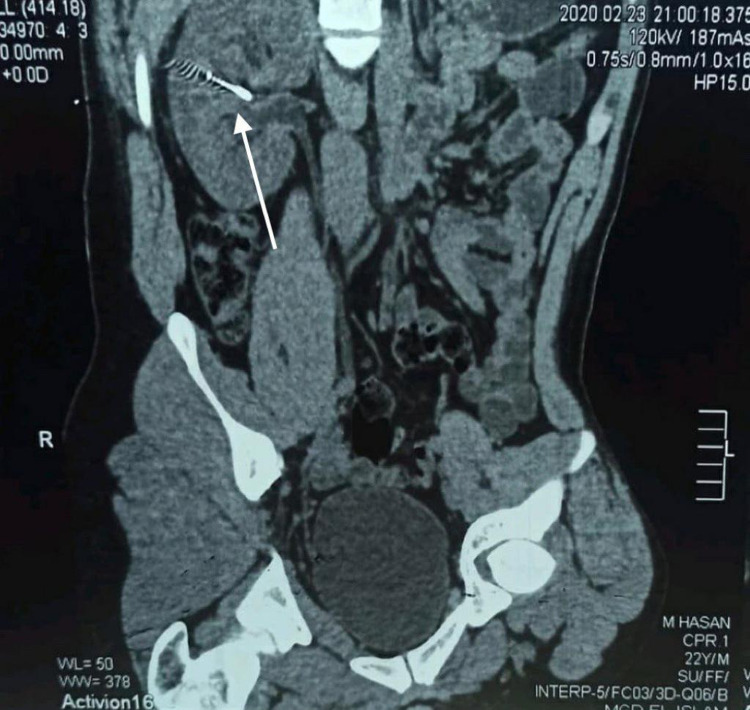
Abdominal CT without contrast shows a 2-inch nail within the right kidney.

**Figure 3:**
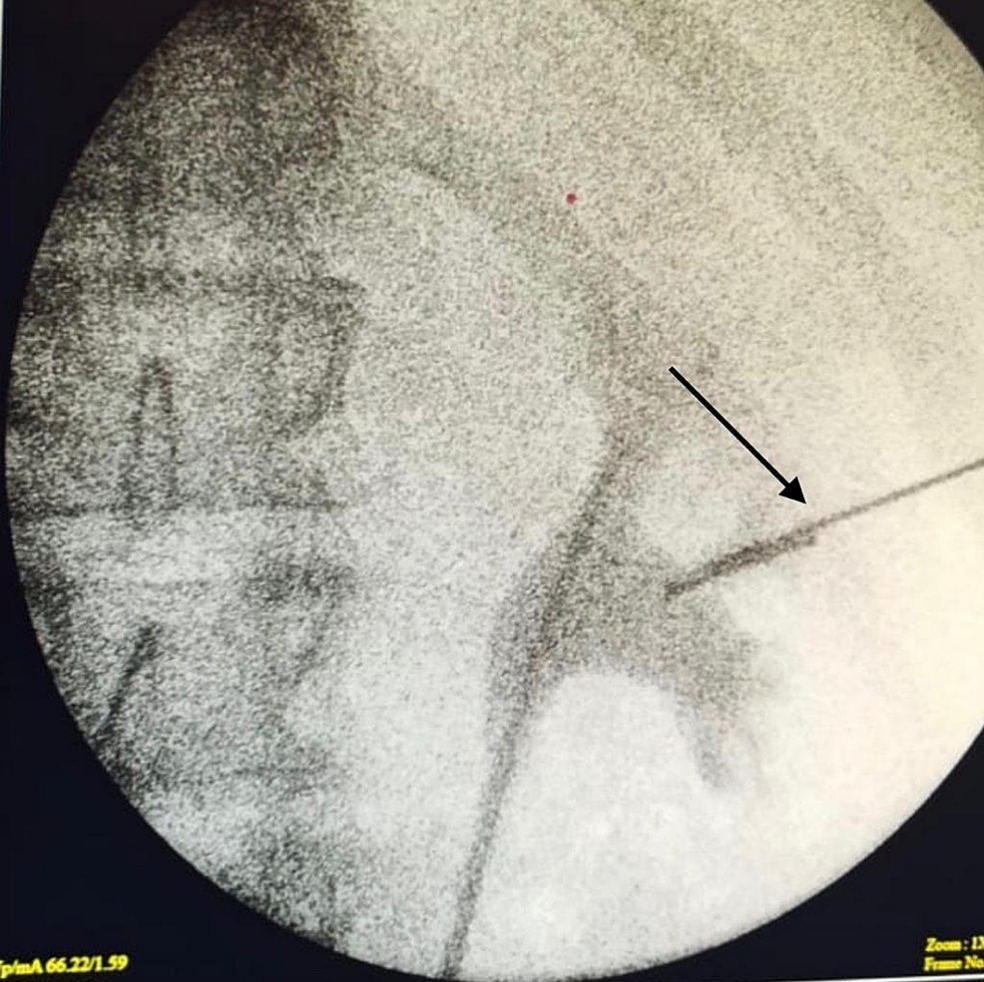
Intraoperative fluoroscopy.

**Figure 4:**
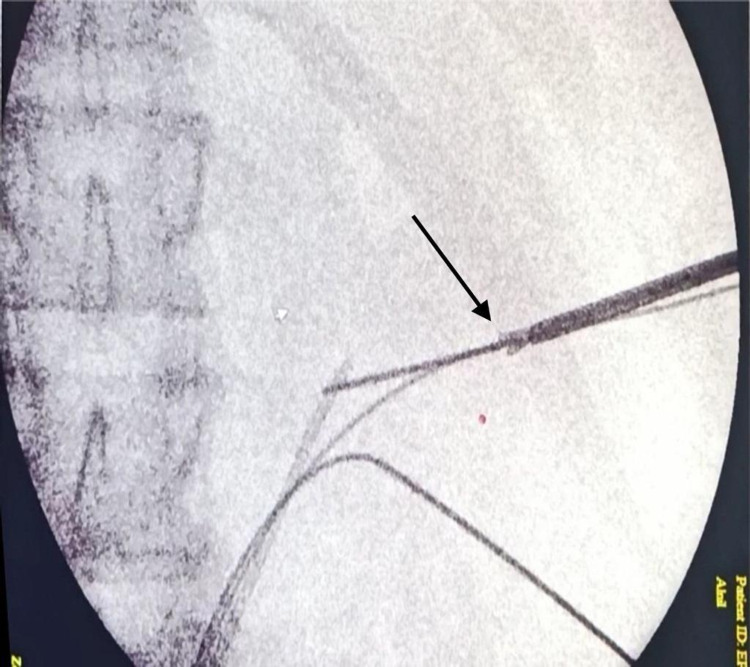
Extraction of the nail by the ureteroscopy forceps with fluoroscopic guidance.

We present a case of a 22-year-old carpenter with an unremarkable medical history. While he was working, one of his coworkers was holding an electric nail gun and accidentally fired a nail that penetrated the patient’s right flank. The patient immediately went to the nearest primary health care unit. He was hemodynamically stable and complained of pain at the site of the injury with mild hematuria. In the primary care unit, an abdominal X-ray scan was done, which confirmed the presence of a metallic nail inside the patient’s flank (Figure 1). The patient was referred to a tertiary care center where he was operated on by general surgeons with fluoroscopy image intensifier guidance. They failed to extract the nail, as it was deep and unreachable. Postoperatively, an abdominal CT without contrast was done, which confirmed the presence of a 2-inch nail deep inside the right kidney (Figure 2). The patient was referred to our center for further management. Besides mild right flank pain, which was possibly due to the recent incision, the patient was doing well with no complaint of hematuria. Preoperatively, laboratory findings including hemoglobin and creatinine were within normal range. Contrast-enhanced CT was decided not to be done to minimize unnecessary radiation exposure from repeated imaging. Therefore, a retrograde pyelography was done. We discussed with the patient the possible interventions to extract the nail, including a retrograde extraction by ureteroscopy or antegrade extraction through percutaneous access or an open extraction if the endoscopic maneuvers failed. The possible complications and outcome if the nail was left, were explained. Following perioperative assessment, the patient received general anesthesia in the operation room. Initially, the patient was placed in a lithotomy position, and a guidewire was inserted into the left kidney. Following balloon dilatation of the lower ureter, a ureteroscopy was performed but the ureter was tight in the middle section, and an open-tipped 6 Fr ureteric catheter to the right renal pelvis and 16 Fr urethral catheter were inserted. The patient was placed in the standard prone position. A retrograde contrast injection under fluoroscopy was done to determine the entry point for the percutaneous extraction (Figure 3). The right kidney was high and was accessed through a supracostal approach to the middle calyx, with a subcostal approach to the lower calyx as an alternative approach. Both were secured by a guidewire going down to the right ureter. The middle calyceal tract was dilated by fascial dilators to 10 Fr, followed by the insertion of a rod guide mounted with a 14 Fr Amplatz renal dilator with its sheath. After dilatation of the tract, an antegrade ureteroscopy was performed with manipulation of the nail to direct its tip toward the access sheath. The renal parenchyma surrounding the nail was friable. Extraction of the nail by the ureteroscopy forceps was done under direct endoscopic vision with fluoroscopic guidance with two guidewires and two punctures. One guidewire was used as a safety guidewire and the other as a standby if we had failed access. (Figure 4). A big tail nephrostomy tube 12 Fr was inserted and fixed at the right renal pelvis to monitor the urine output for 24 hours. In the recovery room, a chest X-ray was done, which revealed no abnormalities. The postoperative period was uneventful, and the patient was discharged. A week later, the patient attended the clinic and was vitally stable with clear urine. Three months postoperatively, the patient was followed-up with a bedside ultrasound and renal function tests, both of which were unremarkable, and he had no complaint or complications.

## Discussion

Following the primary survey and hemodynamic stabilization, a thorough history and physical examination should be performed to determine the mechanism, location, and extent of the renal injury. Laboratory tests, including urinalysis, hematocrit, and creatinine, are essential to identify the presence of hematuria, blood loss, and renal function impairment. Imaging is required in all renal injuries regardless of the type. It is performed primarily to determine the grade of the injury and to evaluate the contralateral kidney for other abnormalities or associated injuries. An intravenous contrast-medium-enhanced CT remains the modality of choice for most renal trauma cases. There are multiple options in the management of renal injuries which include operative and nonoperative interventions. The choice depends on the mechanism and the grade of the injury. Previously, surgical exploration was indicated in almost all cases of penetrating renal injuries; however, recent studies have found that the majority of penetrating renal injuries can be managed conservatively. Moolman et al. reported that 60% of patients with a penetrating renal injury were managed conservatively without the need for surgical intervention [4]. Despite the benefits associated with conservative management, certain cases require intervention; for example, a hemodynamically unstable patient and the presence of a foreign body that must be removed [5]. In this case, the patient had a nail within the right kidney that had to be extracted to avoid possible complications, including infection, encrustation, and stone formation. The nail was extracted via percutaneous access.

## Conclusions

Although multiple challenges were encountered, they were handled efficiently and the patient had an excellent outcome. Percutaneous extraction was used to successfully manage this case, in which the nail was extracted using ureteroscopy forceps under the direct endoscopic vision and fluoroscopic guidance. Since there are no standard guidelines in which penetrating renal trauma should be managed. This unique case and its management may provide valuable information in managing patients with penetrating renal injury.
